# The Revolving Door of Adenovirus Cell Entry: Not All Pathways Are Equal

**DOI:** 10.3390/pharmaceutics13101585

**Published:** 2021-09-29

**Authors:** Davor Nestić, Ksenija Božinović, Isabela Pehar, Rebecca Wallace, Alan L. Parker, Dragomira Majhen

**Affiliations:** 1 Division of Molecular Biology, Ruđer Bošković Institute, 10000 Zagreb, Croatia; Davor.Nestic@irb.hr (D.N.); ksenija.bozinovic@irb.hr (K.B.); Isabela.Pehar@irb.hr (I.P.); 2 Division of Cancer and Genetics, School of Medicine, Cardiff University, Cardiff CF14 4XN, UK; WallaceR1@cardiff.ac.uk (R.W.); ParkerAL@cardiff.ac.uk (A.L.P.)

**Keywords:** adenovirus, endocytosis, vectors, gene therapy

## Abstract

Adenoviruses represent exceptional candidates for wide-ranging therapeutic applications, from vectors for gene therapy to oncolytics for cancer treatments. The first ever commercial gene therapy medicine was based on a recombinant adenovirus vector, while most recently, adenoviral vectors have proven critical as vaccine platforms in effectively controlling the global coronavirus pandemic. Here, we discuss factors involved in adenovirus cell binding, entry, and trafficking; how they influence efficiency of adenovirus-based vectors; and how they can be manipulated to enhance efficacy of genetically modified adenoviral variants. We focus particularly on endocytosis and how different adenovirus serotypes employ different endocytic pathways to gain cell entry, and thus, have different intracellular trafficking pathways that subsequently trigger different host antiviral responses. In the context of gene therapy, the final goal of the adenovirus vector is to efficiently deliver therapeutic transgenes into the target cell nucleus, thus allowing its functional expression. Aberrant or inefficient endocytosis can impede this goal, therefore, it should be considered when designing and constructing adenovirus-based vectors.

## 1. Introduction

Adenoviruses (AdVs) can cause mild health problems, like acute respiratory, gastrointestinal, and ocular infections, but have no oncogenic potential in humans [1]. To ensure viral progeny, AdVs need to efficiently infect permissive cells that will enable both adenoviral entry and replication of DNA. The life cycle of AdVs can be simply divided into several stages: binding to a receptor expressed on a cell surface, entry into the cell by endocytosis, endosomal escape, trafficking through the cytoplasm, and docking at the nuclear pore complex (NPC), followed by nuclear entry of the adenoviral genome. Once adenoviral DNA enters the nucleus, expression of early and late genes allows assembly of new viral particles and their release from the host cell is enabled.

Thanks to their features, like the relative ease with which their genome can be modified as well as relative tolerance towards genetic manipulation, AdVs have been recognized as promising vectors for gene transfer. Today, according to the Wiley database on Gene Therapy Trials Worldwide, AdVs present 17.5% of vectors used in gene therapy clinical trials (https://a873679.fmphost.com/fmi/webd/GTCT; accessed on 10 August 2021). The first ever commercial gene therapy medicine was recombinant adenovirus encoding human p53 tumor suppressor gene, Gendicine, registered for the treatment of head and neck cancers on the market in China [2]. Besides being vectors for gene transfer, AdVs are widely used as vectors for vaccination. Numerous AdV vaccine candidates for treating infectious diseases and cancer are under investigation, with the most important milestone being achieved when European Medicines Agency recently approved three AdV-vector-based vaccines: one against Ebola (Zabdeno; Ad26.ZEBOV [3]) and two against Covid-19 (ChAdOx1 nCoV-19 [4] and Ad26.COV2.S [5]), demonstrating the efficacy of AdV-based vaccines as tools for controlling pandemic outbreaks.

We distinguish the two types of commonly used adenoviral vectors: replication-defective and oncolytic adenoviruses. Due to molecular engineering, replication-defective AdVs cannot replicate in infected cells, however they provide high expression of transgenes with minimum expression of AdV proteins. Unlike replication-defective AdVs (also known as vectors), oncolytic AdVs selectively replicate in and lyse cancer cells, and are, therefore, commonly used vectors in clinical trials for cancer gene therapy [6]. The world first oncolytic virus product was also an adenovirus, Oncorine (H101), an oncolytic adenovirus intended to be used in combination with chemotherapy as a treatment for patients with late-stage refractory nasopharyngeal cancer [7]. Currently, a large number of AdV platforms are being developed for oncology applications, for example, Enadenotucirev, a clinical stage oncolytic AdV with broad potential to target solid tumor carcinomas [8]; DNX-2401; Delta-24-RGD oncolytic AdV, developed as immunotherapeutic treatment for recurrent malignant glioma [9]; and TILT-123, a human AdV5/AdV3 chimeric oncolytic adenovirus encoding for tumor necrosis factor alpha and human interleukin 2 aimed at generating an anti-cancer immune response [10].

To fulfil its role as a vector, an AdV needs to successfully deliver its DNA genome to the host nucleus, a process highly influenced by AdV intracellular translocation. In this review, we discuss which factors are involved in AdV endocytosis, focusing mainly on receptors and endocytic scaffold proteins, as well as the influence of a particular type of endocytosis on AdV intracellular trafficking.

### 1.1. Human Adenoviruses

**Figure 1:**
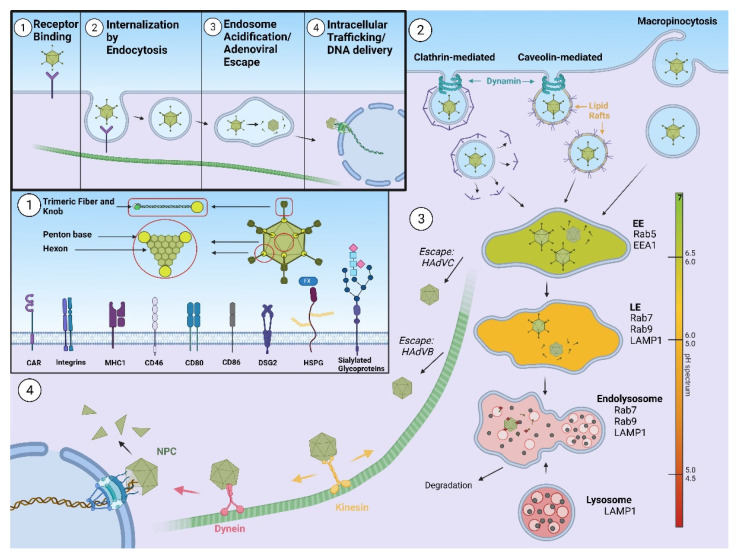
Schematic representation of different steps in adenovirus infection, ultimately leading to delivery of the viral genome to the host cell nucleus. 1. Adenovirus basic structure and various possible binding receptors. 2. Internalization of the virion by different mechanisms of endocytosis. 3. Virion trafficking within vesicles and endosome acidification, followed by virion escape. 4. Docking at the nuclear pore complex and delivery of the adenoviral genome. Abbreviations: CAR – coxsackie and adenovirus receptor; MHC1—major histocompatibility complex class 1; DSG2—desmoglein 2, HSPG—heparan sulfate proteoglycans, FX—factor X, EE—early endosome, LE—late endosome, EEA1—early endosome antigen 1, LAMP1—lysosomal-associated membrane protein 1, HAdVC—human adenovirus subgroup C, HAdVB—human adenovirus subgroup B, NPC—nuclear pore complex.

Human adenoviruses (HAdVs) are non-enveloped double stranded DNA viruses with icosahedral capsids of approximately 90 nm in diameter and mass of ~150 megadaltons [11]. HAdVs consist of 13 structural proteins that form the outer capsid of icosahedral geometry and the inner core, where double-stranded DNA is bound with the core proteins. Major building blocks of the HAdV capsid are hexon and penton. There are 240 copies of the hexon trimer, and 12 pentons comprising an extended fiber protein non-covalently attached to the penton base protein. The fiber protein is composed of tail, shaft, and knob domains, the latter containing several loops. In addition, capsids comprise minor proteins IIIa, VI, VIII, and IX [12]. HAdVs are classified into seven subgroups, A–G [13], according to hemagglutination and serum neutralization reactions or by genome sequencing and bioinformatics analysis (International Committee on Taxonomy of Viruses—ICTV). HAdV infection starts with binding to the primary receptor at the cell surface. Primary receptor usage differs between different HAdVs serotypes. After initial attaching to the primary receptor, HAdV binds αv integrins on the cell surface through an RGD peptide sequence present in the penton base of the virus [14]. This interaction triggers internalization of HAdV, as well as a number of signaling events that lead to virus cell entry within the endosomal compartment [15]. Partial disassembly of the capsid occurs prior to membrane penetration, however the escape of incoming adenoviruses from endosomes is dependent on the viral membrane lytic protein VI, whose lytic activity is pH-independent [16,17]. Once liberated from the endosome, the fibreless HAdV capsid is transported towards the nucleus along a network of microtubules [18] and motor proteins, such as dynein (unidirectional movement) or kinesin (bidirectional) [19,20]. Final docking of the HAdV capsid occurs at the nuclear pore and the HAdV genome enters the nucleus [21], where it remains episomal. A scheme of HAdV cell entry is presented on Figure 1.

### 1.2. Role of the Fiber Receptors’ Interactions in Human Adenovirus Cell Entry

Contact between an HAdV and a cell is achieved through interactions of adenoviral capsid proteins with receptors expressed on the cell surface. Different HAdV types bind to different receptors on target cells and the presence of specific receptors on certain cell types defines HAdV tropism. Additionally, HAdV receptors differ in the ability to allow HAdV attachment and/or internalization. To date, many molecules on the cell surface have been shown to enable HAdV binding: coxsackie and adenovirus receptor (CAR), CD46, desmoglein-2, sialic acid, heparan sulfate proteoglycans (HSPG), integrins, GD1a glycan, major histocompatibility complex (MHC) class I, CD80, CD86, scavenger receptor, and others [22]. In addition, molecules like lactoferrin [23] and coagulation zymogens [24] can bridge receptor-independent HAdV binding to the target cell.

The earliest studies of HAdV intracellular trafficking indicated that different serotypes exhibit different trafficking pathways within the host cell. Depending on the serotype, HAdV capsids either accumulate in the lysosomes (human adenovirus type 7, HAdV7) or are trafficked directly to the nuclear envelope (human adenovirus type 5, HAdV5) [25]. Currently, the majority of information known about HAdV intracellular trafficking comes from studying HAdVs belonging to subgroups C (human adenovirus type 2, HAdV2 and HAdV5) and B (human adenovirus type 35, HAdV35). These two subgroups use different primary receptors, CAR (HAdV5) and CD46 (HAdV35), directing them toward different internalization mechanisms. Subgroup B serotypes accumulate in lysosomes, whereas subgroup C serotypes traffic rapidly to the nuclear envelope, revealing different kinetics of endosomal escape. Subgroup C serotypes’ optimum pH for membrane lysis matches the one of early sorting endosomes, while subgroup B serotypes’ optimum matches the pH of late endosomes or lysosomes [26]. Thus, HAdV intracellular trafficking is clearly determined by the initial interaction with the cellular receptor.

To fulfil their function as vectors for gene therapy, HAdV need to successfully deliver the gene of interest to the nucleus of target cells. A major limitation of HAdVs when considered as vectors for gene therapy is their inability to accomplish gene delivery to specific cells of interest. This can be circumvented by targeting HAdV towards molecules specifically expressed on the target tissue. Targeting strategies employing manipulation of the HAdV genome among others include peptide incorporation to the HI loop and C-terminus of the knob domain, serotype chimerism, fiber/knob replacement, and combinatorial approaches—chimeric HAdV composed of fiber/knob domains from alternative serotypes, fiber-xenotyped HAdV vectors displaying fibers from nonhuman AdV species, and knobless HAdV fiber–fibritin fusion [6]. Genetic incorporation of peptides into the HVR (hypervariable region) of hexon and C-terminus of the pIX are also possible [27].

Importance of the fiber–receptor interaction was corroborated in the experiments when swapping the fiber protein between different serotypes (known as pseudotyping) resulted in altered intracellular trafficking of modified HAdV. Namely, Ad5f7, a chimeric vector composed of the HAdV5 capsid with the fiber replaced by the HAdV7 fiber, exhibited intracellular trafficking characteristics more similar to those of HAdV7 rather than HAdV5, suggesting that substitution of the HAdV7 fiber had a significant impact on intracellular trafficking of the HAdV5 capsid and delivery of the HAdV5 genome to the nucleus [28]. Similarly, recycling of the chimeric capsids comprising the HAdV5 long fiber and HAdV35 knob toward the plasma membrane has been described. While Ad5 virus efficiently escaped from the endosomal environment early after infection, Ad5/35L virus entered late endosomal/lysosomal compartments and remained there for at least 2 h. Some of the Ad5/35L virions reached the nucleus in late endosomes/lysosomes, while other virions appeared to be transported in a retrograde manner and deposited in or around adhesion foci at the cell surface. Taken together, these data demonstrate that even though only the fiber knob domain was different between Ad5 and Ad5/35L vectors, they selected different intracellular trafficking routes [29]. The dominant role of the fiber—receptor interaction for HAdV endocytosis was further described in the context of heparan sulphate proteoglycans (HSPGs) and coagulation factor X (FX). The vitamin-K-dependent carboxylation/gamma-carboxyglutamic (Gla) domain of the FX protein is responsible for Ca^2+^-dependent phospholipid membrane binding, and therefore, plays crucial roles in the interaction of FX with its binding partners [30]. The importance of FX binding is clear when AdVs are systemically administered. It was shown that FX binds to the HAdV5 hexon via an interaction between the FX Gla domain and hypervariable regions of the hexon surface, thus directing HAdV5 towards a CAR-independent alternative infection pathway involving HSPGs [31]. Contrary to HAdV5, in the presence of FX, the serotype 35 fiber-pseudotyped vector HAdV5F35 accumulated in the late endosomal compartment, resulting in a delay in its vesicular release and nuclear import. Furthermore, HAdV5F35 was released in significant amounts in the extracellular medium via exocytosis, resulting in lower numbers of particles reaching the nucleus [32]. Of note, presence of FX increased transduction efficiency of subgroup D fiber-pseudotyped viruses [33], indicating that dominance of fiber over FX is dependent on fiber serotype and receptor engagement. The interaction of HAdV with the blood coagulation factors and the resulting influence on liver transduction and HAdV biodistribution has been previously reviewed [34].

Conversely, targeting AdGFP-QM10, an HAdV5 vector expressing a green fluorescent protein (GFP) as a reporter gene and harboring a GHPRQMSHVY peptide (QM10) insertion within the fiber shaft, to an alternative entry pathway on airway cells seemed to confer upon the vector a significant advantage in terms of gene transfer efficiency in a number of cell lines. Virions of AdGFP-QM10 were endocytosed in higher numbers than virions of the control vector and were directed to a compartment different from the early endosomes targeted by members of subgroup C. AdGFP-QM10 was found to accumulate in late endosomal and low-pH compartments, suggesting that QM10 acted as an endocytic ligand of the lysosomal pathway [35].

More recently, HAdV5 retargeted towards folate receptor alpha (FRα), which is highly expressed on ovarian cancer cells, was evaluated for virotherapy application. Unfortunately, despite increased binding efficiency, the recombinant vectors with FRα-targeting peptide incorporated in the HI loop of HAdV5 fiber failed to efficiently transduce target cells via FRα due to defective intracellular trafficking following entry via FRα. This indicates that whilst the FRα targeting peptides used in this study may have potential for applications for targeted drug delivery, they require additional refinement for targeted virotherapy applications [36].

Interventions aimed at introducing novel tropism can modify cell entry of newly designed AdV vectors, potentially resulting in impaired endocytosis or intracellular trafficking of adenoviral vectors. Even though it has been generally accepted that AdV fiber protein mediates attachment of virions to cells and that fibers dissociate during endocytic uptake [37], it is obvious that both fiber and knob alone, i.e., interaction with the receptor, likely modulate intracellular trafficking as well. For example, by directing the AdV vector to a degradative environment, like lysosomes, and thus modifying the presentation of the virus to host immune recognition systems, and as such presenting an obstacle in gene therapy. Therefore, before considering HAdV targeting through ligand modification, one should study receptor–ligand interaction in order to ensure compatibility with viral uptake and intracellular trafficking. 

There is no doubt HAdV binding to primary receptor influences downstream endocytosis pathways, however the role of the penton base RGD domain should not be disregarded in this process. It has been found that for both CAR- and CD46-interacting vectors, deletion of the RGD motif in the penton base reduces the rate of virus particle internalization into human cells. Moreover, RGD motif-dependent interactions with cellular integrins support efficient virus escape from endosomes [38,39,40]. However, deleting the RGD motif did not change transduction efficiency of HAdV5 in rat hepatocytes and endothelial cell lines in vitro [41]. It has been shown that wild-type (wt) and ΔRGD HAdV5 viruses behave differently in 3D and 2D cultures, with ΔRGD viruses consistently showing a lower relative transduction in 3D cultures when compared to wt viruses. In 2D cultures, this was receptor- and cell line-dependent [42]. In vivo, the penton RGD motif has been shown to be involved in the splenic accumulation of HAdV5 particles following intravascular delivery. ΔRGD HAdV5 exhibits decreased splenic uptake in immune cells, corresponding with decreased levels of innate antiviral cytokines, indicating that this mutation may be beneficial in limiting antiviral responses when using HAdV-based vectors for intravascular applications [43,44].

## 2. Different Endocytosis Pathways and Human Adenovirus Receptors

Endocytosis is the process of taking a particle or substance from outside of the cell towards the inside in the form of an endocytic vesicle. Extracellular ligands that bind to a specific cell surface receptor are internalized, along with their receptor/s, inside of the newly formed endocytic vesicles. Internalized receptor–ligand complexes undergo various fates, influenced by the nature of the vesicle. Many receptors release their ligand in the acidic milieu of the late endosome. Afterwards, the receptors are usually sorted into vesicles that recycle them to the plasma membrane, while the ligands are sorted into vesicles that fuse with lysosomes. In this endocytic pathway, the released ligands are degraded by lysosomal enzymes [45]. Except for being indispensable for the cell, endocytosis is also hijacked by viruses for the cell entry. Both non-enveloped and enveloped viruses share the same main steps and routes of viral entry, which begin with the attachment to the cell surface receptors and end with intracellular delivery of viral genome. After binding to receptors, viruses can utilize two main paths to enter the cell, the endocytic and non-endocytic routes. Unlike enveloped viruses, which can internalize host cells via both non-endocytic fusion and the endocytic route, non-enveloped viruses, such as AdVs can use only receptor-mediated endocytosis for efficient cell entry. The most commonly considered endocytic route used by viruses is transport in clathrin-coated vesicles or pits, but non-clathrin-coated pits, macropinocytosis, or caveolae are also used [46]. Besides efficient binding to a specific receptor, a sufficient number of adenoviral particles per cell (multiplicity of infection, MOI) is also required for efficient cell entry. For example, it has been suggested that AdV endosomal escape is dependent on having an activated macropinocytic pathway, however this may be relevant only at high MOI [47].

The role of clathrin and caveolin, two major endocytosis proteins, has been described in internalization and sorting of some HAdV receptors. It has been reported that basolateral sorting of the CAR, which anatomically is located at the tight junctions [48], proceeds through interaction of a canonical YXXΦ motif with the clathrin adaptors AP-1A and AP-1B [49], and that AP-1B knockdown in MDCK cells missorted CAR from recycling endosomes to the apical surface of the cell [50]. Yet, it has been reported that CAR is localized to a lipid-rich microdomain similar to that of the low-density lipoprotein receptor (LDLR) but distinct from caveolin-1-containing caveolae and GPI-linked proteins [51]. Residence in a lipid-rich domain provides a mechanism that allows CAR to interact with other cell adhesion proteins and function as a HAdV receptor. A study focusing on testing whether CAR could leave lipid microdomains upon HAdV binding and internalize in a clathrin-dependent pathway demonstrated that CAR internalization in neuronal cells was lipid microdomain-, actin-, and dynamin-dependent, but clathrin-independent. In addition, internalization of CAR was followed by degradation in lysosomes [52]. 

Clathrin involvement was reported also for another HAdV receptor, CD46. It has been described that ligand binding and the cell type determine whether CD46 is internalized by clathrin-coated pits or macropinocytosis. For instance, in nonlymphoid cells, CD46 is constitutively internalized via clathrin-coated pits and is recycled to the cell surface while cross-linking of CD46 and measles virus elicits pseudopodial extension similar to macropinocytosis [53]. Cell-type-dependent internalization of CD46 receptor is not the only example of how cell type influences endocytic route. We will mention in the following chapters how the same AdV can use different endocytic routes depending on host cell type. Both clathrin and caveolin have been described as implicated in integrin internalization mechanisms. It has been reported that the clathrin-mediated endocytic machinery facilitates the endocytosis of RGD–integrin β3 clusters [54], while αvβ5 integrin was found to cluster to flat clathrin lattices, dynamic actin-controlled hubs for clathrin-mediated endocytosis and signaling of specific receptors [55,56].

### 2.1. Clathrin-Mediated Endocytosis

Early studies suggested that one of the first steps in the infectious cycle of HAdV2 is internalization by the receptor-, coated pit-, and vesicle-mediated pathway [57]. Later, it was reported that HAdV uptake was increased by overexpression of wild-type Rab5 and decreased by dominant-negative Rab5, indicating a role for Rab5 in HAdV entry [58]. Soon after, clathrin-mediated endocytosis, the best described receptor-mediated endocytosis regulated by Rab5, became known as the major uptake pathway used by HAdV. 

Clathrin-mediated endocytosis can be divided into three basic steps: (1) assembly of clathrin coat, (2) invagination of clathrin-coated pits, and (3) releasing of clathrin-coated vesicles into the cytosol [59]. A coated pit is assembled due to interaction of adapter proteins, clathrin, and transmembrane receptors (cargo). We distinguish different endocytic adapters that are crucial for early stages of clathrin-mediated endocytosis: adapter protein-2 (AP-2), epidermal growth factor receptor substrate 15 (Eps15), Eps15-interacting protein (Epsin), disabled protein 2 (Dab2), clathrin assembly lymphoid myeloid leukemia protein (AP180/CALM), and clathrin-associated sorting protein (CLASP) [60]. Subsequently, joining of different accessory and regulatory proteins that function as scaffolds, cargo recruiters, membrane curvature generators, or sensors contributes to stabilization, maturation, and dismissal of clathrin-coated vesicles, which are then sorted and transported to various destinations in the cell [61]. Clathrin-mediated endocytosis is also dynamin-dependent. Dynamin is a protein with GTPase activity and acts as scissors when it comes to clathrin-coated vesicle separation from membrane [62].

Studies aimed at investigating whether HAdV2-mediated endocytosis was clathrin-dependent have utilized various dominant negative forms of key regulators of clathrin-mediated endocytosis, namely expression of dominant negative clathrin hub, eps15, and discovered that impeding clathrin-mediated endocytosis restricted HAdV2 cell entry, establishing that clathrin-mediated endocytosis is the major entry pathway of HAdV2 [47]. Besides HAdV2, HAdV5 wild type and the non-infectious temperature-sensitive Ad2-ts1 mutant [63] also use clathrin-dependent endocytosis to internalize into epithelial cells. Just as in the case of HAdV2, HAdV5 and Ad2-ts1 cell entry was dependent on clathrin adaptors Eps15 and AP180. However, while HAdV2 and HAdV5 reached the cytosol in a Rab5-independent manner, Ad2-ts1 depended on Rab5 for endocytosis and transport to late endosomes and lysosomes. These data suggest that clathrin and dynamin-dependent HAdV trafficking to early endosomes share a common set of factors, but can differ in their requirements of Rab5 [64]. Even though the kinetics of Ad2-ts1 uptake into cells is comparable to HAdV2, suggesting similar endocytic uptake mechanisms, it has been reported that unlike HAdV2 and HAdV5, Ad2-ts1 uptake does not require CALM, a protein that controls clathrin-mediated endocytosis and membrane transport between endosomes and the trans-Golgi-network. Contrary to Ad2-ts1, which takes a route to late endosomes/lysosomes, HAdV2 requires CALM for infectious endocytosis or endosomal escape. This suggests that CALM directly or indirectly supports cytosolic escape of HAdV2 from early endosomes or trans-Golgi-network membranes [65]. Clathrin-dependent endocytosis of HAdV2 was shown to be cholesterol-dependent since cholesterol extraction from the plasma membrane inhibited rapid HAdV2 endocytosis, endosomal escape, and nuclear targeting [66].

As stated in the previous section, HAdV retargeting is employed when target cells lack a primary receptor, often CAR, or in cases when the aim is to avoid CAR-mediated cell entry. A commonly used strategy involves abrogation of CAR-mediated cell entry coupled with the introduction of targeting towards desired molecule. Examples of such targeting include the use of bispecific molecules, for example targeting Fcγ receptor 1 of hematopoietic cells using an adaptor comprising the extracellular CAR domain (which binds to the adenoviral fiber knob protein, preventing interaction with the native primary receptor, CAR) and the Fc portion of a human immunoglobulin G (CARex-Fc) for retargeting purposes. This targeting resulted in AdV aggregates, which were internalized to the cells by phagocytosis. Escape of Fcγ-R-targeted AdV from phagosomes was clathrin-dependent, i.e., clathrin knockdown inhibited viral escape from phagosomes, indicating that Fcγ-R-mediated transduction of hematopoietic cells requires early stages of clathrin-mediated endocytosis. Thus, the authors proposed a cooperative interaction of clathrin-mediated endocytosis and phagocytosis triggering phagosomal lysis and infection [67].

Use of clathrin-mediated endocytosis was reported also for non-CAR binding HAdV. Cellular entry of human adenovirus type 37 (HAdV37), belonging to subgroup D, in human corneal epithelial cells occurs primarily by clathrin-mediated endocytosis, but in a dynamin-independent manner. Further, it was found that HAdV37 entry in these cells depends on modification of actin, but does not require key components of canonical clathrin-mediated endocytosis, such as epsin, endosomal acidification, or early endosome antigen 1 (EEA1) [68]. It has been shown that HAdV37 uses GD1a glycan as a cellular receptor [69], where the use of sialic acids appears a common mechanism evolved for HAdV causing epidemic keratoconjunctivitis [70], but may also bind αvβ1 and α3β1 integrins for infection of human corneal cells [71].

When used at high multiplicity of infection, human adenovirus type 3 (HAdV3) of subgroup B, which uses desmoglein-2 as primary receptor [72], can internalize via a clathrin-independent mechanism in the absence of fluid-phase stimulation. Still, the majority of the HAdV3 particles were found in large endocytic vesicles and only a minor fraction was found in clathrin-coated invaginations or vesicles, suggesting that clathrin has a minor role in HAdV3 endocytosis. It is likely that clathrin is required for endosomal escape of HAdV3 [73]. Of note, the internalization of a virus-like particle derived from the HAdV3, called the adenoviral dodecahedron (Dd), in leukemic cells involved clathrin-mediated energy-dependent endocytosis and strongly correlated with the expression of αvβ3 integrin [74]. 

The importance of clathrin-mediated endocytosis for functional infection with subgroup C HAdVs was indirectly confirmed in hematopoietic cells. It has been proposed that nonpermissivity of these cells, especially B lymphocytes, could be the consequence of inefficient subgroup C HAdV particle uptake caused by inadequate localization of clathrin, in addition to low CAR expression. It was noted that in epithelial cells the clathrin is observed in association with membranes, while in the hematopoietic cell lines it is found predominantly in the cytoplasm. Thus, altered clathrin-coated pit endocytosis could explain the weak subgroup C HAdV uptake in B cells [75]. Expression of HAdV receptors other than CAR or integrins was not assessed in this study.

### 2.2. Lipid Rafts and Caveolin-Mediated Endocytosis

Over the years, as we learned more and more about HAdV biology, it has become increasingly obvious that adenovirus uses multiple different pathways to enter the cell. Thus, it has been reported that, in certain cases, HAdVs can also use lipid rafts and caveolae as routes of cell entry.

When discussing lipid rafts and caveolae, it is important to define these two terms accurately. Lipid rafts are dynamic low-density membrane microdomains (10–200 nm) that are rich in free cholesterol and glycosphingolipids [76]. Although lipid rafts are membrane domains, the plasma membrane is not the only location where lipid rafts occur; it is now known that lipid rafts reside also in intracellular membranes, as well as in extracellular vesicles. Among other functions, lipid rafts play roles in signal transduction, cholesterol homeostasis, receptor activation, and forming sorting platforms for targeted protein and lipid trafficking [77]. Caveolae are specialized lipid rafts microdomains that consist of small plasma membrane invaginations (60–80 nm in diameter) at the cell surface. The inner layer of caveolae is composed of proteins called caveolins, while the outer layer consists of protein cavins. Three main caveolin proteins found in caveolae of mammalian cells are caveolin-1 (cav-1), caveolin-2 (cav-2), and muscle-specific caveolin-3 (cav-3), with cav-1 being the main structural component of caveolae since it has capacity to induce formation of caveolae. Caveolae are directly involved in the internalization of membrane components, extracellular ligands, bacterial toxins, and several non-enveloped viruses [78]. In the context of HAdV, endocytosis that occurs in lipid rafts can be both caveolin-dependent or -independent. 

There are two scenarios that describe fate of cargo after detachment of caveolae from the plasma membrane. In the first scenario, caveolae fuse with an early endosome and follow the classical endosomal–lysosomal degradation pathway. In the second scenario, caveolae fuse with the caveolar endosome and are targeted specifically to endoplasmic reticulum (ER), therefore, cargo will avoid the classical degradational endocytic pathway [79]. Both scenarios happen frequently, and the composition of the plasma membrane within the caveolar vesicle is critical for the function of caveolae in intracellular membrane trafficking, which, in fact, determines if caveolae will fuse with the caveolar endosome or the classical early endosome [80]. 

Studies on HAdVs that use lipid raft/caveolin-mediated endocytosis for cell entry have not been as extensive as studies on clathrin-mediated endocytosis. The first evidence of caveolae-mediated HAdV cell entry was shown in mature plasmocytic cells, which, in contrast to B lymphocytes, are efficiently infected by HAdVs despite aberrant clathrin-mediated entry [75]. Efficient infection of mature plasmocytic cells with HAdV5 is not the result of clathrin-coated pit endocytosis, since this process is inefficient in these cells, but rather caveolae/lipid-raft-mediated entry, since it has been shown that HAdV5 colocalizes with caveolae/lipid rafts in plasmocytes. Moreover, inhibiting caveolae endocytosis by depletion of cholesterol or expression of dominant negative caveolin-1 in these cells reduced HAdV5 infectivity. Thus, it is possible that HAdVs use caveolae endocytosis when a clathrin-dependent pathway is not available [81]. The authors of this study also proposed that targeting HAdVs towards caveolae may represent an alternative approach to enhancing uptake in most hematopoietic cells. They employed this hypothesis in their subsequent work. By investigating the endocytic pathway of chimeric adenoviral vectors harboring fibers constituted of the N-terminal domain of HAdV2 and the knob domain of a bovine virus, BAdV4, HAdV2/BAdV4, they demonstrated that a CAR-independent AdV vector can use lipid raft/caveolae endocytosis in CAR-negative cell lines. Uptake of HAdV modified in such a way led to non-altered intracellular trafficking without endosomal retention. This indicated that by forcing HAdVs to take advantage of a non-clathrin pathway, it is possible to compensate for the deficiency in endosomolysis associated with the use of some fiber-modified adenoviral constructs [82].

Above, we discussed how HAdV37 uses primarily clathrin-mediated endocytosis to enter human corneal epithelial cells. This same virus can enter human keratocytes through caveolae. It has been shown that cholesterol-rich lipid raft microdomains and caveolin-1 are essential for HAdV37 entry into primary human corneal fibroblasts. Cholesterol depletion, using methyl-β-cyclodextrin, profoundly reduced viral infection and HAdV37 was identified in caveolin-1-rich endosomal fractions after infection. In their study, HAdV2, a non-corneal pathogen, appeared to also utilize the caveolar pathway for entry into A549 cells but failed to infect corneal cells entirely, indicating virus- and cell-specific tropism [83]. These findings suggest both a redundancy and cell specificity in mechanisms of adenoviral entry.

### 2.3. Macropinocytosis

Another endocytic pathway, macropinocytosis, has been described as involved in infectious entry of subgroup B HAdV. Macropinocytosis involves nonspecific intake of extracellular material in vesicles called macropinosomes, whose size varies between 0.2 and 10 μm [84]. During growth-factor-induced macropinocytosis, actin polymerizes and cell membrane remodels into macropinosome-forming protrusions. Macropinocytosis is independent of dynamin-2, and the main factors regulating macropinocytosis are PAK-1, Arf6, and Rho factors of the GTPase family—Rac1 and Cdc42 [85].

It has been described that HAdV3, unlike HAdV5, uses dynamin-independent endocytosis for rapid infectious entry into epithelial and hematopoietic cells. HAdV3 endocytosis, which spatially and temporally coincided with enhanced fluid-phase uptake, was sensitive to macropinocytosis inhibitors targeting F-actin, protein kinase C, the sodium–proton exchanger, and Rac1, but not Cdc42. Within the infection pathway, HAdV3 was found in macropinosomes that also contained the CD46, αv integrins, and the C-terminal binding protein 1 of E1A (CtBP1). The authors also observed that infection of hematopoietic cells with HAdV5 was independent of CtBP1 but sensitive to clathrin siRNA, further confirming that HAdVs of different serotypes can infect the same cells in a different manner, not only receptor wise but also endocytosis wise [73]. 

Macropinocytosis is a cell entry pathway for another subgroup B member, low seroprevalent HAdV35. Infectious entry of HAdV35 into HeLa cells, human kidney HK-2 cells, and normal human lung fibroblasts, requires CD46, integrins, PKC, a sodium/proton exchanger, actin, Rac1, Pak1, and CtBP1, but not heparan sulfate and, variably, the large GTPase dynamin. HAdV35 infection is independent of expression of the carboxy-terminal domain of AP180, but is inhibited by the F-actin inhibitor jasplakinolide, the Pak1 inhibitor IPA-3, or amiloride (EIPA), which are known inhibitors of macropinocytosis. Similar observations were made using small interfering RNAs against factors driving macropinocytosis, including the small GTPase Rac1, Pak1, or the Pak1 effector C-terminal binding protein 1 (CtBP1). Like HAdV3, HAdV35 colocalized with fluid-phase markers in large endocytic structures that were positive for CD46, αv integrins, and also CtBP1 [86]. These data extend earlier observations for HAdV3 and establish macropinocytosis as an infectious pathway for subgroup B HAdVs in epithelial and hematopoietic cells. 

Since both HAdV3 and HAdV35 can use CD46 as a receptor, one can hypothesize that CD46 directs HAdVs towards macropinocytosis, indicating that fiber–receptor interaction plays a major role in this decision. This is further corroborated by the fact that while the HAdV3 enters epithelial and hematopoietic cells using macropinocytosis, a viral-like particle composed of only 12 copies of HAdV3 penton base uses clathrin-mediated endocytosis [74]. 

While subgroup B members use macropinocytosis as a cell entry pathway, subgroup C HAdVs enter the cell mostly by clathrin-mediated endocytosis, however they can trigger the macropinocytosis along the way. It has been reported that binding of HAdV2 to epithelial cells triggers macropinocytosis, coincident with the clathrin-mediated viral uptake. Nevertheless, this HAdV2-induced macropinocytosis did not depend on viral endocytosis, nor was it needed for viral uptake. The authors suggested that macropinosome formation and leakage is crucial for viral escape from endosomes and for infection [47]. How HAdV infection triggers macropinocytosis has been further studied during infection of the acinar epithelial cells of the lacrimal gland (LGAC) with HAdV5. Analysis of fluorescein isothiocyanate (FITC)-dextran uptake and time-lapse video microscopy of green fluorescent protein (GFP)-actin ruffling showed that HAdV5 could stimulate macropinocytosis in LGAC, a process that could also be elicited by the fiber and knob but not penton base. Additional studies suggested that macropinocytosis is important for efficient HAdV5 transduction but that it does not participate directly in HAdV5 entry. Taken together, these findings suggest that HAdV5 internalization into LGAC is through a unique fiber-dependent pathway that may involve CAR and HS-GAGs, rather than the penton base–integrin-mediated endocytic mechanism seen in other cell models so far investigated [87].

### 2.4. Intracellular Trafficking

From all that has been discussed so far, one can conclude that HAdVs use multiple different pathways for cellular entry. These paths will inevitably direct them to different cytoplasmic compartments from which they need to escape to reach the nucleus. It is generally accepted that the functional infection pathway of HAdVs involves entry and, subsequently, escape from the early endosome. In human epithelial cells, HAdV5 is internalized in to early endosomes [64] and is primarily found at the nuclear membrane with only a small proportion colocalized with LAMP1, a marker of late endosome [88]. Besides viral factors that are involved in escape from the endosome, such as the previously mentioned protein VI, there are also cellular molecules that regulate HAdV’s exit from the endosome. Additionally, different routes of administration can impact the cell entry of HAdVs when used in vivo, however this is beyond the scope of this review.

**Figure 2:**
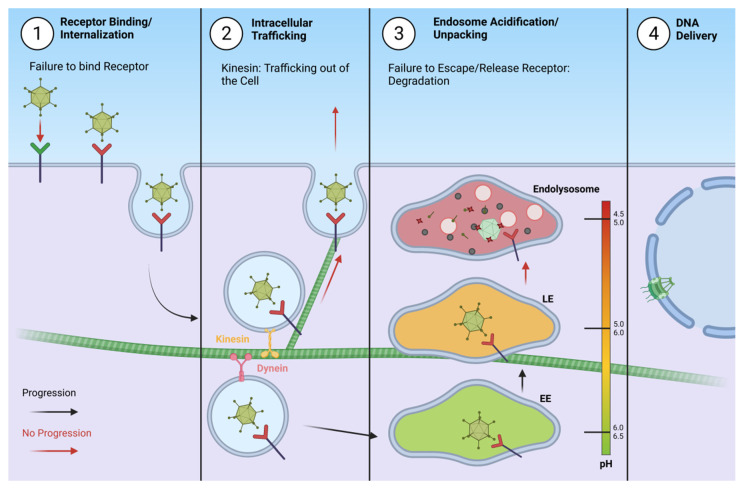
Unsuccessful delivery of the adenovirus genome may occur at several steps during the process of infection. 1. Binding an inadequate receptor that does not allow for internalization. 2. Vesicle trafficking via kinesin may lead to exocytosis of the virion. 3. Inability to escape the endosome due to firm binding of the receptor may cause accumulation and eventually lead to degradation in the endolysosome. 4. Unsuccessful infection prevents genome delivery and production of progeny virions.

Unsuccessful delivery of the AdV genome may occur at several steps during the process of infection, therefore, proper AdV intracellular trafficking is important when AdVs are considered as vectors, both for gene therapy and vaccination. Aberrant intracellular trafficking can be caused by binding to an inadequate receptor, exocytosis of the virion due to trafficking via kinesin, or inability to escape the endosome and eventual degradation in the endolysosome. Except aberrant genome delivery, unsuccessful infection also prevents production of progeny virions. An outline of steps that can go wrong during AdV infection is presented on Figure 2.

Early studies of AdV cell entry suggest that the interaction of αvβ5 integrin with HAdV2 penton base facilitates the subsequent step of virus internalization into the cell. These studies provided evidence for the involvement of a cellular receptor in virus-mediated membrane permeabilization and proposed a novel biological role for αvβ5 integrin in the infectious pathway of an HAdV, beyond the binding step [89]. Later, it was demonstrated that the intracellular domain of the integrin β5 subunit specifically regulates HAdV-mediated membrane permeabilization and gene delivery [90].

The role of endosomes was also studied as a mechanism by which alveolar macrophages can avoid infection by HAdV5 during clearance. It has been reported that transcription factor PU.1 uncouples HAdV5 internalization from infection by confining it to the endosome/lysosomal pathway. This study demonstrated that PU.1 blocked HAdV-mediated endosome lysis and that HAdV5 colocalized entirely with endosomal markers in PU.1-expressing cells. Increased expression of PU.1 caused accumulation of HAdV5 in LAMP1-positive vesicles and decreased transduction efficiency of the virus. Finally, PU.1 redirected adenoviral trafficking in a manner similar to that caused by bafilomycin, but without changing endosome acidification. Instead, PU.1 reduced expression of integrin β5, known to be crucial in HAdV-mediated endosome lysis [91].

Integrin αvβ5 proved to have an important role also in transduction efficiency of HAdV5, containing a pan integrin engaging RGD4C retargeting peptide in the HI loop of the fiber knob protein. By using an HEp-2 (human laryngeal carcinoma) cell model with different expression of αvβ3/5 integrin, we demonstrated that whilst internalization of RGD-modified HAdV5 is considerably increased in an HEp2-β3 transfectant expressing a very high amount of β3, transduction is reduced, consistent with reduced expression of β5 integrin. In this cell clone, the expression of β5 integrin becomes limited in transduction, presumably owing to its crucial role in membrane permeabilization [39].

Different HAdV serotypes utilize different endocytic compartments and show different intracellular trafficking, which, ultimately, all result in productive infection. Indeed, it was shown that HAdV7 from subgroup B firstly accumulated in the cytoplasm of A549 cells and afterwards occupied acidic compartments (pH 5) over the first 2 h, with a gradual shift toward neutrality by 8 h post-infection. HAdV7 partially colocalized with α2-macroglobulin and late endosomal and lysosomal marker proteins, including Rab7, mannose-6-phosphate receptor, and LAMP1. The pH optimum for membrane lysis by HAdV7, as well as a chimeric HAdV5 capsid that expressed the HAdV7 fiber (Ad5f7) [28], was pH 5.5 [92]. Thus, the native trafficking pathway for HAdV7 involves residence in late endosomes and lysosomes, with information encoded in the HAdV7 fiber acting as a pH-dependent trigger for membrane lysis and escape to the cytosol. Therefore, the retention of HAdVs in late endosomal compartments does not necessarily need to be detrimental in regards to HAdV function. Additionally, it can influence the way the host immune system recognizes HAdVs, which has to be kept in mind when considering the design of HAdVs as vectors for therapeutic gene transfer applications.

HAdVs are the most deployed viral platform for cancer applications. It has been shown that changes occurring in tumor cells during development of resistance to anticancer drugs can be beneficial for HAdV-mediated transgene expression. By using an in vitro model consisting of a parental cell line, human laryngeal carcinoma HEp2 cells, and a cisplatin-resistant clone CK2, we investigated the cause of increased HAdV5-mediated transgene expression in CK2 as compared to HEp2 cells. We demonstrated that the primary cause of increased HAdV5-mediated transgene expression in CK2 cells is not modulation of receptors on the cell surface or change in HAdV5 attachment and/or internalization, but is rather the consequence of decreased RhoB expression. While in CK2 cells at 30 min post-infection, HAdV5 was located at the very border of the nucleus, in HEp2 cells, most of the viruses were still scattered in the cytoplasm. Thus, we proposed that RhoB plays an important role in HAdV5 post-internalization events and, more particularly, in HAdV5 intracellular trafficking [93]. Since RhoB has been shown to regulate endosome transport by promoting actin assembly on endosomal membranes [94], one can imagine that manipulation of RhoB could interfere with the endosomal retention of HAdV5.

## 3. Host Innate Immune Response and Human Adenovirus Escape from Endosome

Due to their pathogen-associated molecular patterns (PAMPs), HAdVs can activate innate immune response. HAdV PAMPs are recognized by pattern-recognition receptors (PRRs), among which the most prominent are Toll-like (TLRs), AIM2-like (ALRs), and NOD-like receptors (NLRs) [95]. Following internalization into the endosome, adenoviral DNA can be recognized by TLR9. Involvement of TLR9 in immune activation has been reported for CAR- and CD46-utilizing HAdV vectors [96]. HAdV detection by NLRP3 containing inflammasome is dependent on HAdV penetration of endosomal membranes, the release of the lysosomal protease cathepsin B into the cytoplasm, and the production of reactive oxygen species. Inflammasome activation by HAdV5 also leads to NLRP3 and cathepsin B-dependent, but caspase-1-independent, necrotic cell death, resulting in the release of the proinflammatory molecule HMGB1. Thus, rupture of cathepsin-enriched lysosomes during HAdV5 cell entry serves as a danger signal leading to the release of proinflammatory mediators [97]. Additionally, it has been shown that HAdV-induced inflammasome activation can be influenced by HAdV endosomal sorting. HAdV5 vectors possessing human adenovirus type 16 (HAdV16) knob domains (HAdV subgroup B, CD46-utilizing), Ad5f16, have shown greater localization to cathepsin-enriched lysosomes prior to endosomal escape, which led to enhanced cytosolic release of cathepsins and augmented reactive oxygen species production compared to HAdV5, even though both viruses penetrate endosomal membranes with similar efficiency [98]. 

It has been proposed that HAdV37 can use different pathways to enter cells of different origin which could account for a relative paucity of proinflammatory gene expression upon infection with this virus [68,83]. In the case of HAdV37, the role of dynamin-2, a protein that allows detachment of the endocytic vesicle from the plasma membrane, in triggering innate immune response has also been studied. Overexpression of dynamin-2 prior to viral infection led to a statistically significant increase in CXCL10 and CXCL8, while downregulating dynamin-2 led to statistically significant reductions in CCL2, CCL5, CXCL1, CXCL11, IL-1ra, IL-6, MIF, and Serpin E1 [99]. Connection of virus localization and cytokine expression after HAdV infection was reported also for HAdV35 and HAdV26, both used as vaccine platforms [100,101]. Of note, engagement of CD46 as a cellular receptor appears to dampen the host immune response against the infected cell through downregulation of C/ERPbeta, a situation that could have implications when CD46 binding AdVs, like HAdV35, are considered for gene delivery and vaccine development [102]. It has been shown that HAdV35 and HAdV26 accumulate in the late endosomal compartment more extensively than HAdV5 at 2 to 8 h following infection and that innate immune stimulation by all HAdV vectors was sensitive to inhibitors of endosomal acidification, cathepsin B, and caspase 1 [103]. The role of endosomal escape in HAdV-triggered innate response was seen also with Ad2-ts1 mutant, which is defective in endosomal escape [63]. While HAdV2 induced rapid phosphorylation of p38 and ERK, as well as a significant cytokine response, Ad2-ts1 failed to activate p38 or ERK and induced only a limited cytokine response [104]. 

Contrary to the neutralizing effect observed in epithelial cells, HAdV5 infection in the presence of antiviral antibodies significantly increased FcR-dependent viral internalization in macrophages. In direct correlation with the increased viral internalization, antiviral antibodies amplified the innate immune response to HAdV5, as determined by the expression of NF-κB-dependent genes, type I IFNs, and caspase-dependent IL-1β maturation. Confocal microscopy revealed subversion of natural tropism and targeting toward LAMP1-positive phagolysosomes in HAdV5-infected macrophages in the presence of antiviral antibodies, but not in epithelial cells. This skewing toward phagolysosomes amplified innate immune response [105].

Taken together, these data suggest that route of entry of endosomal trafficking and escape of HAdVs is a critical factor in the induction of innate immune response. Stalling HAdVs in one of the endocytic vesicles can induce activation of the innate immune system that otherwise would not happen. In addition, a dynein-mediated unidirectional movement towards the perinuclear microtubule minus end results in faster capsid transport towards the nucleus, therefore, kinesin-mediated bidirectional movement could slow down the HAdV and possibly contribute to HAdV stalling [20]. Aberrant recognition by innate immune sensors can be not only deleterious for potential vectors but can also cause undesired host immune response. Therefore, correct intracellular trafficking of the HAdV vector is needed not only to assure functionality in terms of successful delivery of transgene, but also in order not to provoke unintended immune response and, hence, increase safety of vectors intended for therapeutic applications.

## 4. Outlook

HAdV infection is a complex process involving several steps: binding to a primary receptor, internalization, escape from the endosome, intracellular trafficking and finally genome delivery to the nucleus. In this review we described how factors involved in almost all those stages can be orchestrated by cellular endocytosis pathways. Endocytosis can change HAdV receptors’ expression or influence their recycling, thus, impeding their availability on the cell surface. Aberrant endocytosis itself can change the internalization procedure of an HAdV and force it to use another method of cell entry. This can influence not only transgene expression but can also trigger different host immune response which in some instances can be undesired, but in others might be beneficial. Regardless of the fact that AdVs are considered not to be very dangerous, adenoviral infection is a significant cause of mortality in the immunocompromised individual, so understanding their means of infection and entry may also be critical at the level of developing new antivirals to treat such patients.

In summary, the reasons for the different intracellular traffic of HAdVs of different serotypes may be different: (1) binding to different receptors on the cell surface triggers different signals for HAdVs to enter the cell; (2) HAdVs enter cells with different types of endocytosis; (3) during intracellular trafficking, different HAdVs serotypes are found in different types of endosomes, from which they may have different exit efficiencies; (4) HAdV travel depends on motor proteins and others. When considering the use of HAdV-based vectors for translational therapeutic applications, these represent key considerations when designing constructs for optimal transgene expression in target cells.
